# Association between risk of dementia and very late-onset schizophrenia-like psychosis: a Swedish population-based cohort study

**DOI:** 10.1017/S0033291721002099

**Published:** 2023-02

**Authors:** J. Stafford, J. Dykxhoorn, A. Sommerlad, C. Dalman, J. B. Kirkbride, R. Howard

**Affiliations:** 1 Division of Psychiatry, University College London, London, UK; 2 Camden and Islington NHS Foundation Trust, London, UK; 3 Department of Global Public Health, Karolinska Institutet, Stockholm, Sweden; 4 The Center for Epidemiology and Social Medicine (CES), Region Stockholm

**Keywords:** Dementia, old age psychiatry, psychiatric epidemiology, psychosis, schizophrenia, very late-onset schizophrenia-like psychosis

## Abstract

**Background:**

Although the incidence of psychotic disorders among older people is substantial, little is known about the association with subsequent dementia. We aimed to examine the rate of dementia diagnosis in individuals with very late-onset schizophrenia-like psychosis (VLOSLP) compared to those without VLOSLP.

**Methods:**

Using Swedish population register data, we established a cohort of 15 409 participants with VLOSLP matched by age and calendar period to 154 090 individuals without VLOSLP. Participants were born between 1920 and 1949 and followed from their date of first International Classification of Diseases [ICD], Revisions 8–10 (ICD-8/9/10) non-affective psychotic disorder diagnosis after age 60 years old (or the same date for matched participants) until the end of follow-up (30th December 2011), emigration, death, or first recorded ICD-8/9/10 dementia diagnosis.

**Results:**

We found a substantially higher rate of dementia in individuals with VLOSLP [hazard ratio (HR): 4.22, 95% confidence interval (95% CI) 4.05–4.41]. Median time-to-dementia-diagnosis was 75% shorter in those with VLOSLP (time ratio: 0.25, 95% CI 0.24–0.26). This association was strongest in the first year following VLOSLP diagnosis, and attenuated over time, although dementia rates remained higher in participants with VLOSLP for up to 20 years of follow-up. This association remained after accounting for potential misdiagnosis (2-year washout HR: 2.22, 95% CI 2.10–2.36), ascertainment bias (HR: 2.89, 95% CI 2.75–3.04), and differing mortality patterns between groups (subdistribution HR: 2.89, 95% CI 2.77–3.03).

**Conclusions:**

Our findings demonstrate that individuals with VLOSLP represent a high-risk group for subsequent dementia. This may be due to early prodromal changes for some individuals, highlighting the importance of ongoing symptom monitoring in people with VLOSLP.

## Background

Although non-affective psychotic disorders typically have their first onset in adolescence or early adulthood (Kessler et al., 2007), recent population-based evidence suggests a second peak of incidence after 60 years old, particularly in women (Stafford, Howard, & Kirkbride, 2018; Stafford, Howard, Dalman, & Kirkbride, 2019). This is referred to as very late-onset schizophrenia-like psychosis (VLOSLP) in those aged over 60 years old (Howard, Rabins, Seeman, & Jeste, 2000). There is ongoing debate about the aetiology of VLOSLP and its relationship with neurodegeneration and dementia (Brodaty, Sachdev, Koschera, Monk, & Cullen, 2003; Vahia et al., 2010; Van Assche, Morrens, Luyten, Van de Ven, & Vandenbulcke, 2017). Understanding this issue is critical to providing insight into the aetiologies of VLOSLP and dementia, which could then inform clinical practice.

Data on the association between VLOSLP and cognitive decline are, however, sparse, and most studies in this area have been limited by small, unrepresentative samples, and cross-sectional designs (Van Assche et al., 2017). Two longitudinal, population-based studies have focused on VLOSLP and dementia, reporting elevated risk of dementia in people with VLOSLP, but these studies had relatively short follow-up periods (maximum 7 years) (Kørner, Lopez, Andersen, & Kessing, 2008; Kørner, Lopez, Lauritzen, Andersen, & Kessing, 2009). This is problematic because dementia neuropathology may develop over decades (Bateman et al., 2012; Villemagne et al., 2013), with symptoms emerging up to 12 years before dementia diagnosis (Amieva et al., 2008), meaning that it is not possible to determine whether the observed association is because VLOSLP is a cause of dementia, or an early symptom. Long follow-up periods are required to fully characterise the relationship between VLOSLP and dementia and reduce the risk of protopathic bias.

We examined the rate of subsequent dementia diagnosis in a large, Swedish, population-based cohort of individuals with VLOSLP and an age-matched comparison group without VLOSLP. We hypothesised that individuals with VLOSLP would have a higher rate of subsequent dementia diagnosis, and a shorter time-to-dementia-diagnosis than those without VLOSLP. We considered death as a potential competing risk, given higher mortality rates in those with psychotic disorders (Hayes, Marston, Walters, King, & Osborn, 2017), including VLOSLP (Talaslahti et al., 2015). However, we hypothesised that findings would not be explained by this competing risk or by ascertainment bias related to previous contact with health services in those with VLOSLP. Finally, given the lack of previous data in this area, we expected that the risk of dementia associated with VLOSLP would be similar across demographic subgroups, which we assessed by testing interactions between VLOSLP and sex, education level, and family liability for psychotic disorder.

## Methods

### Study design and population

We used Psychiatry Sweden data, a nationwide population register linkage for mental health research. We established a matched cohort design, including individuals living in Sweden born between 1920 and 1949 who were first diagnosed with an International Classification of Diseases (ICD; Eight, Ninth and Tenth Revision codes (WHO, 1992), online Supplementary Table S1) non-affective psychotic disorder in the NPR at age 60 years or older with no prior dementia diagnosis.

We constructed an age-period-cohort-matched comparison group (matched within the same birth year), without a diagnosis of non-affective psychotic disorder in the NPR (10 matches per person with VLOSLP). Matched participants were required to be alive, living in Sweden, and without a dementia diagnosis on the date of their matched individual's VLOSLP diagnosis. The date of VLOSLP diagnosis, or that of an individual's matched participant, was used as the start of follow-up. Participants were followed until first recorded diagnosis with dementia in the NPR, death, emigration from Sweden, or the end of the follow-up period on 31 December 2011, whichever was earliest.

### Outcome

Our main outcome was first dementia diagnosis recorded in the National Patient Register (NPR), which includes inpatient (1973–2011) and outpatient records (2001–2011) (ICD-8/9/10 codes listed in online Supplementary Table S2).

### Exposure

The primary exposure was VLOSLP diagnosis (ICD codes listed in online Supplementary Table S1) in the NPR (earliest date of ascertainment: 1st January 1980).

### Covariates

Data on age, sex, and region of birth were obtained from the Swedish Register of the Total Population. Region of birth was broadly categorised as follows: Sweden, Finland, other Nordic, other European, and other countries. Disposable income at age 60 years was obtained from the Longitudinal Integration Database for Health Insurance and Labour Market Studies (LISA) and grouped into quartiles based on all cohort members with disposable income from all sources (employment, welfare receipts, savings, investments). We obtained data on educational attainment from the LISA, grouped as pre-high school, high school, and post-high school. Familial liability of psychotic disorder was obtained by linking participants to their biological children using the Multigenerational Register and identifying whether any of these children had received a diagnosis of non-affective psychosis (as per codes in online Supplementary Table S1) in the NPR.

### Missing data

Individuals with missing data, limited to disposable income and educational attainment, were excluded from the cohort prior to matching (5.4%; online Supplementary Table S3).

### Statistical analysis

First, we presented descriptive statistics of the cohort. Second, we used Cox regression to examine dementia rates in those with and without VLOSLP. We initially examined univariable associations between covariates and dementia diagnosis, assessing model fit using Akaike's information criterion (AIC), with lower scores indicating better fit. Next, we added variables with the lowest AIC values individually into a model including sex as an *a priori* confounder and the matching variable. We retained covariates in a forward-fitting model if they improved model fit, assessed via likelihood ratio test (LRT). We fitted and tested interactions (via LRT) between VLOSLP and sex, education level and family liability for psychotic disorder. We assessed the proportional hazards assumption using Schoenfeld residuals plots and tests.

Third, we used accelerated failure time (AFT) models to estimate how much quicker those with VLOSLP were diagnosed with dementia relative to the non-VLOSLP group, expressed via time ratios. We compared the fit of different distributions for the AFT error term (including exponential, Weibull, log-normal or log-logistic) via AIC. We re-ran our crude and best-fitting model, as identified from Cox regression, in the AFT parameterization. Fourth, we assessed the potential impact of differential patterns of mortality on the association between VLOSLP and dementia using Fine and Gray competing risks regression (Fine & Gray, 1999).

To examine the potential effect of misdiagnosis of dementia as VLOSLP, we conducted sensitivity analyses with washout periods, excluding those who were diagnosed with dementia within six months, one year, or two years from VLOSLP diagnosis, and their matched comparison participants, regardless of their outcome status. We conducted a second sensitivity analysis to investigate whether any observed association between VLOSLP and dementia could have been attributable to possible increased detection of dementia in the VLOSLP group. This may have arisen, if for example, they had more contact with health services as a result of VLOSLP than the matched comparison group, leading to more opportunities for detection of dementia. To test this, we divided the matched comparison group into those with and without a recorded inpatient or outpatient diagnosis for any condition in the year either side of cohort entry. If the rate of dementia was only higher in the VLOSLP group relative to the non-VLOSLP group without a recent diagnosis (but not relative to the non-VLOSLP group with a diagnosis during this time), this may suggest that findings were due to ascertainment bias.

## Results

### Cohort characteristics

Our cohort consisted of 15 409 participants diagnosed with VLOSLP and 154 090 matched participants without VLOSLP. During follow-up, 13 610 (8%) individuals were diagnosed with dementia (VLOSLP: 17.8%; non-VLOSLP: 7.1% (χ^2^(1) = 2200, *p* = <0.001)). Median age-at-first-dementia-diagnosis was younger in the VLOSLP group (76 years, interquartile range (IQR): 72–81) than the non-VLOSLP group (82 years, IQR: 78–86; Mann−Whitney *z* = 37.4, *p* ⩽ 0.001). Compared with the non-VLOSLP group, participants with VLOSLP were more likely to be women (60.6% *v.* 53.8%), have a lower education level (pre-high school education: 59.1% *v.* 53.2%), have a lower disposable income at age 60 years old (lowest quartile: 36.6% *v.* 26.1%), have family liability for psychotic disorder (5.3% *v.* 2.5%), and were less likely to be Swedish-born (85.5% *v.* 90.9%) (all *p* ⩽ 0.001; Table 1).

**Table 1.** Cohort characteristics in those with and without VLOSLP

Characteristic	No VLOSLP (*N* = 154 090)	VLOSLP (*N* = 15 409)	
	Median (IQR)	Median (IQR)	Mann−Whitney test
Age-at-diagnosis with dementia [inter-quartile range (IQR)]	82 (78–86)	76 (72–81)	*p* = <0.001
	*N* (%)	*N* (%)	χ^2^ test
Dementia group			χ^2^(1) = 2200.00, *p* = <0.001
Dementia	10 866 (7.05)	2744 (17.81)	
No dementia	143 224 (92.95)	12 665 (82.19)	
Sex			χ^2^(1) = 259.41, *p* = <0.001
Men	71 224 (46.22)	6078 (39.44)	
Women	82 866 (53.78)	9331 (60.56)	
Educational attainment			χ^2^(2) = 239.24, *p* = <0.001
Pre-high school	81 994 (53.21)	9111 (59.13)	
High school	49 827 (32.34)	4614 (29.94)	
Post-high school	22 269 (14.45)	1684 (10.93)	
Disposable income at age 60			χ^2^(3) = 2100.00, *p* < 0.001
Lowest (1)	40 236 (26.11)	5642 (36.61)	
2	36 892 (23.94)	4935 (32.03)	
3	39 747 (25.79)	3007 (19.51)	
Highest (4)	37 215 (24.15)	1825 (11.84)	
Family liability for non-affective psychotic disorder			
Yes	3878 (2.52)	815 (5.29)	χ^2^(1) = 399.95, *p* < 0.001
No	150 212 (97.48)	14 594 (94.71)	
Region of birth			
Sweden	139 999 (90.86)	13 173 (85.49)	χ^2^(4) = 497.18, *p* < 0.001
Other Nordic	2573 (1.67)	333 (2.16)	
Finland	4819 (3.13)	852 (5.53)	
Other European	5321 (3.45)	858 (5.57)	
Other	1378 (0.89)	193 (1.25)	

### Association between VLOSLP and subsequent dementia

**Fig. 1.**
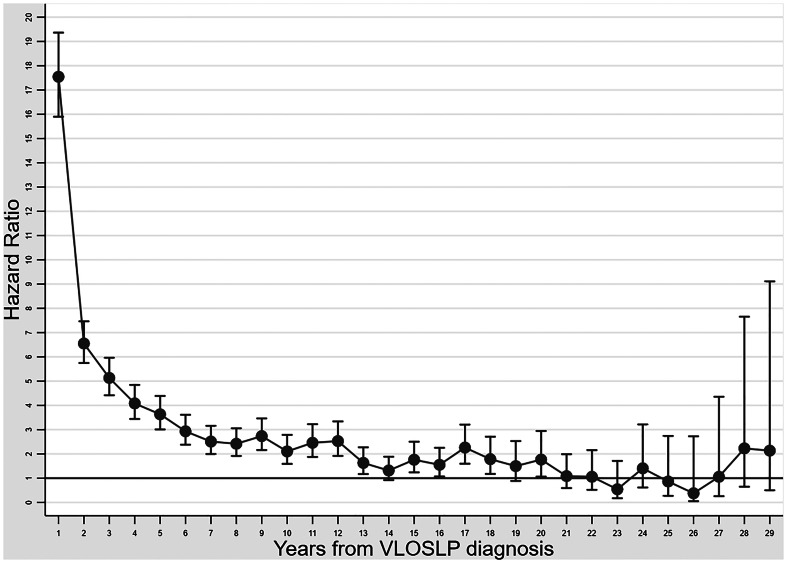
Association between very late-onset schizophrenia-like psychosis and dementia during follow-up. Dementia HRs with 95% confidence intervals.

In Cox regression models, compared with the non-VLOSLP group, we found a higher rate of dementia in participants with VLOSLP [fully adjusted hazard ratio (HR): 4.22, 95% confidence interval (95%CI) 4.05–4.41; Table 2], after adjustment for sex, education level, disposable income, region of birth and family liability for psychotic disorder. There was evidence of non-proportional hazards for the VLOSLP exposure, but not for other covariates (online Supplementary Table S4). Further exploration of this issue (Fig. 1) suggested that the dementia HR associated with VLOSLP was highest in the first year after VLOSLP diagnosis, although rates of dementia remained higher in the VLOSLP group for up to 20 years of follow-up.

**Table 2.** Association between VLOSLP and incident dementia

Variable	Model 1^a^	Model 2^b^	6-month washout^b^ *N* excluded: 8756 (5%)	1-year washout^b^ *N* excluded: 11 451 (7%)	2-year washout^b^ *N* excluded: 15 356 (9%)
*N* included (%)	169 499 (100)	169 499 (100)	160 743 (95)	158 048 (93)	154 143 (91)
Cox proportional hazards regression HRs for dementia by VLOSLP status (95% CI)					
VLOSLP (ref: no VLOSLP)	4.21 (4.04–4.39)	4.22 (4.05–4.41)	3.12 (2.97–3.27)	2.76 (2.62–2.91)	2.22 (2.10–2.36)
Fine and Gray regression subdistribution HRs for dementia by VLOSLP status (95% CI)					
VLOSLP (ref: no VLOSLP)	2.90 (2.80–3.03)	2.89 (2.77–3.03)	2.07 (1.97–2.18)	1.82 (1.73–1.92)	1.45 (1.37–1.53)
Weibull AFT model time ratios for dementia by VLOSLP status (95% CI)					
VLOSLP (ref: no VLOSLP)	0.25 (0.24–0.27)	0.25 (0.24–0.26)	0.41 (0.40–0.43)	0.46 (0.44–0.48)	0.56 (0.54–0.58)

a Model 1: Adjusted for matching variable.

b Model 2: Adjusted for VLOSLP group, sex, education level, family liability for non-affective psychotic disorder, disposable income at age 60, region of birth, and matching variable.

The association between VLOSLP and dementia varied by socio-demographic subgroup (online Supplementary Table S5), with evidence of effect modification between VLOSLP status and sex (LRT, *p* = <0.001), education level (LRT, *p* = <0.001), and family liability for psychotic disorder (LRT, *p* = 0.01), respectively. Thus, in the VLOSLP group, we observed a lower risk of dementia among women compared with men (HR: 0.86, 95% CI 0.79–0.92), whereas this pattern was reversed in the comparison group (HR: 1.10, 95% CI 1.05–1.14). In the comparison group, individuals with the highest educational attainment had a lower rate of dementia (HR: 0.93, 95% CI 0.86–0.99), which was not found in the VLOSLP group (HR: 0.98, 95% CI 0.85–1.12). By contrast, in the VLOSLP group, the rate of dementia was lower in those with the lowest educational attainment (HR: 0.80, 95% CI 0.73–0.87), which was not observed in the comparison group (HR: 1.03, 95% CI 0.99–1.08). Family liability for psychotic disorder was associated with a higher rate of dementia in the comparison group (HR: 1.21, 95% CI 1.09–1.35), but not in the VLOSLP group (HR: 0.94, 95% CI 0.80–1.11).

Median time-to-dementia-diagnosis in those without VLOSLP was 9.1 years (IQR: 4.3–14.7), relative to 1.9 years (IQR: 0.4–6.0) in those with VLOSLP. In AFT models, a Weibull distribution for baseline survivorship provided best fit to the data (online Supplementary Table S6), and after full adjustment for covariates, a VLOSLP diagnosis was associated with a time ratio of 0.25 (95%CI 0.24–0.26) (Table 2), indicating that time-to-dementia-diagnosis was 75% shorter in this group than those without VLOSLP.

### Mortality as a competing risk

Although mortality was higher in those with VLOSLP (fully adjusted HR: 2.85, 95% CI 2.78–2.91; online Supplementary Table S7), the rate of dementia remained significantly higher in the VLOSLP group in a fully adjusted Fine and Gray regression, incorporating death as a competing risk [sub-distribution hazard ratio (SHR): 2.89, 95% CI 2.77–3.03] (Table 2).

### Sensitivity analyses

Using washout periods of 6-months, 1-year or 2 years to exclude participants whose dementia may have been misdiagnosed as VLOSLP (and their matched participants without VLOSLP), we found some attenuation in elevated dementia rates in the VLOSLP group, but these remained substantially higher than in the non-VLOSLP group in both fully adjusted Cox regression (2-year washout HR: 2.22, 95% CI 2.10–2.36; Table 2) or competing risks regression (SHR: 1.45, 95% CI 1.37–1.53; Table 2).

We conducted a further sensitivity analysis to investigate whether higher rates of dementia in people with VLOSLP were explained by ascertainment bias arising from increased contact with the health system. The results showed that dementia rates were higher in the VLOSLP group relative to both the non-VLOSLP groups who had not received any diagnosis of any condition 12-months either side of cohort entry (HR: 4.90, 95% CI 4.69–5.13), and those who had received a diagnosis during this time (indicative of health service contact) (HR: 2.89, 95% CI 2.75–3.04;Table 3). These results suggest that higher rates of dementia in the VLOSLP group cannot be fully explained by a greater probability of detection of dementia due to greater contact with services.

**Table 3.** Association of VLOSLP with incident dementia: sensitivity analyses to take into account differences in detection by VLOSLP status due to previous contact with health services

Fully adjusted Cox proportional hazards regression				
Variable	HR (95%CI)	6-month washout HR (95%CI) *N* excluded: 8756 (5%)	1-year washout HR (95%CI) *N* excluded: 11 451 (7%)	2-year washout HR (95%CI) *N* excluded: 15 356 (9%)
*N* included (%)	169 499 (100)	160 743 (95)	158 048 (93)	154 143 (91)
VLOSLP (ref: comparison group, no diagnosis near date of entry)	4.90 (4.69–5.13)	3.63 (3.45–3.82)	3.23 (3.06–3.41)	2.61 (2.46–2.77)
VLOSLP (ref: comparison group, any diagnosis near date of entry)^a^	2.89 (2.75–3.04)	2.09 (1.98–2.21)	1.84 (1.73–1.95)	1.47 (1.38–1.56)
Fully adjusted Fine and Gray competing risks regression				
Variable	SHR (95% CI)	6-month washout SHR (95%CI) *N* excluded: 8756 (5%)	1-year washout SHR (95%CI) *N* excluded: 11 451 (7%)	2-year washout SHR (95%CI) *N* excluded: 15 356 (9%)
*N* included (%)	169 499 (100)	160 743 (95)	158 048 (93)	154 143 (91)
VLOSLP (ref: comparison group, no diagnosis near date of entry)	3.08 (2.94–3.22)	2.21 (2.09–2.32)	1.94 (1.84–2.05)	1.54 (1.46–1.64)
VLOSLP (ref: comparison group, any diagnosis near date of entry)^a^	2.52 (2.39–2.66)	1.79 (1.69–1.90)	1.56 (1.47–1.66)	1.24 (1.16–1.32)

Abbreviations: HR, hazard ratio; SHR, subdistribution hazard ratio; VLOSLP, very late-onset schizophrenia-like psychosis.

All analyses are adjusted for**:** VLOSLP group, sex, education level, family liability for non-affective psychotic disorder, disposable income at age 60, region of birth and matching variable.

a Any hospital diagnosis on the year either side of entry into the study (except psychotic disorders or dementia).

To examine the joint effects of possible misdiagnosis and ascertainment bias on our results we re-ran these sensitivity analyses together (Table 3). The rate of dementia remained higher in the VLOSLP group relative to both comparison groups (*v.* participants in the non-VLOSLP group without diagnosis, with a 2-year washout HR: 2.61, 95% CI 2.46–2.77; *v.* participants in the non-VLOSLP group with diagnosis, 2-year washout HR: 1.47, 95% CI 1.38–1.56); similar patterns were present in the competing risk regression model (Table 3).

## Discussion

### Summary of findings

We found a substantially higher rate of subsequent dementia diagnosis among individuals with VLOSLP, who were diagnosed with dementia 75% more quickly than age-period-cohort matched comparison participants without VLOSLP. The relationship between VLOSLP and dementia persisted in our most conservative sensitivity analyses, suggesting that these findings are unlikely to be fully explained by the competing risk of mortality, possible misdiagnosed dementia, or ascertainment bias. Although the association between VLOSLP and dementia was strongest in the first year after VLOSLP diagnosis, the rate of dementia remained higher among the VLOSLP group over up to 20 years of follow-up. Taken together, our findings are consistent with the possibilities that VLOSLP is a prodromal feature of dementia, or that VLOSLP independently confers risk of later dementia via other mechanisms.

### Strengths and limitations

To our knowledge, this is the largest study to date to examine rates of dementia in those with VLOSLP, using a comprehensive, nationwide cohort of people aged over 60 years old, and with the longest follow-up duration (up to 30 years). No previous studies have had sufficiently large samples and long enough follow-up periods to allow adequate detection of incident dementia in those with VLOSLP and characterise the consistency of associations over time. We were also able to consider other potential explanations for findings, including misdiagnosis, ascertainment bias and competing risks. Our use of age-period-cohort matched comparison participants implicitly controlled for several unobserved effects which may have otherwise arisen as a function of these variables, including changes in diagnostic systems over time or recording of dementia in clinical care.

We acknowledge several limitations. While the specificity of dementia diagnoses in the Swedish registers is high, sensitivity is relatively low, with around half of dementia cases in the population not recorded in the registers (Rizzuto et al., 2018). This will have under-estimated the true rate of dementia in the population in this study. However, we would not have expected this bias to have acted differentially by VLOSLP status, except in relation to ascertainment bias as highlighted above, which we examined via sensitivity analyses. Although our sensitivity analysis took into account differences in the number of health service contacts between those with and without VLOSLP, we were unable to fully account for differences in the nature of contacts. It remains possible that our findings partly reflect greater involvement of mental health specialists and increased focus on identifying signs of cognitive decline among the VLOSLP group during follow-up.

A validation study reported an average delay of 5.5 years in recording of dementia diagnoses in the registers (Rizzuto et al., 2018) which may have compromised the precision of estimates of time between VLOSLP and dementia diagnoses. Misclassification between dementia subtypes is common in the NPR (Rizzuto et al., 2018), hence we were not able to examine whether the association varied according to different diagnostic subtypes.

Further, misdiagnosis between schizophrenia and dementia remains a possibility. Pre-existing cognitive deficits are common in schizophrenia (Bora, 2015) and could contribute to misdiagnosis of dementia in some cases. Conversely, early signs of dementia could be mistaken for cognitive deficits and negative symptoms in schizophrenia. Previous studies have shown that comorbidities are often under-detected in people with schizophrenia (Roberts, Roalfe, Wilson, & Lester, 2006; Smith, Langan, Mclean, Guthrie, & Mercer, 2013), possibly due to diagnostic overshadowing, whereby comorbidities are misattributed to psychotic symptoms (Viron & Stern, 2010). Although we conducted a sensitivity analysis to mitigate against misdiagnosis, and we would expect clinicians to be aware of the complexities of dementia diagnoses in the context of psychotic disorders, we cannot fully exclude the possibility of misdiagnosis in either direction. While we adjusted for length of education, we were unable to obtain more detailed information on educational attainment or cognitive functioning from the Swedish registers, which would have allowed greater insight into baseline cognitive function and reserve.

Finally, although we excluded potential participants diagnosed with non-affective psychotic disorders in the registers before 60 years old, register data on psychiatric disorders were only available since 1973 meaning that we were unable to identify participants in either the VLOSLP or non-VLOSLP group who developed a diagnosable psychotic disorder before this date. This may have led us to include some participants with adult-onset psychosis in both the VLOSLP and non-VLOSLP groups. We would expect a higher proportion of these participants to be in the VLOSLP group, hence the accelerated rate of dementia in this group may partly reflect longer-standing psychiatric morbidity; previous studies have shown that adult-onset psychotic disorders are also associated with an increased risk of dementia (Cai & Huang, 2018; Ribe et al., 2015).

### Meaning of findings

Our finding of an elevated rate of dementia diagnosis in individuals with VLOSLP corresponds with an earlier Danish study which found an elevated rate of dementia among people with late-onset schizophrenia (rate ratio (RR): 3.47, 95% CI 2.19–5.5) and VLOSLP (RR: 3.15, 9% CI 1.93–5.14) compared to osteo-arthritis patients (Kørner et al., 2009), and a recent cohort study conducted in Israel also demonstrating an association between VLOSLP and dementia (HR: 2.67, 95% CI 1.82–3.91) (Kodesh et al., 2020). However, both studies were limited by follow-up periods shorter than 5 years.

Previous studies have also demonstrated associations between dementia and younger-onset psychotic disorders (Cai & Huang, 2018). A Danish register study found that schizophrenia was associated with more than 2-fold increased rates of dementia (incidence rate ratio: 2.13, 95% CI 2.00–2.27) (Ribe et al., 2015). In a cohort study of men in Western Australia, Almeida et al. (2018) found an association between dementia and VLOSLP (HR: 2.22, 95% CI 1.74–2.84), and younger-onset psychotic disorders (<65 years HR: 2.73, 95% CI 2.34–3.18).

We have extended previous findings by quantifying time-to-dementia-diagnosis, estimated to be 75% sooner in those with VLOSLP; using longer follow-up to clarify the consistency and persistence of the association; accounting for several potential sources of bias; and examining differences by socio-demographic subgroup. Our results highlight the importance of monitoring cognition and function in patients with VLOSLP, particularly in the first few years following diagnosis. These findings have clinical implications for treatment planning and may warrant reflection in clinical guidelines (Mueller, Thompson, Harwood, Bagshaw, & Burns, 2017).

We observed differential patterns of association between dementia and educational attainment in VLOSLP and non-VLOSLP groups. In the non-VLOSLP group, higher education was associated with lower dementia risk, consistent with evidence regarding cognitive reserve (Sharp & Gatz, 2011). In contrast, for those with VLOSLP, lower educational attainment was associated with a reduced rate of dementia. While we are unsure of the mechanism leading to this finding, one possibility is that individuals with VLOSLP from a lower education group may be less likely to contact services or to have dementia symptoms detected.

The rate of dementia diagnosis was particularly elevated for the VLOSLP group in the first year after VLOSLP diagnosis. As evidenced by sensitivity analyses, this may partly reflect misdiagnosis, and ascertainment bias due to increased contact with health services. However, importantly, the rate of dementia remained higher among the VLOSLP group for up to 20 years of follow-up. This finding is consistent with several explanations. One possibility is that psychotic disorders, including VLOSLP, could increase risk for dementia via factors such as poor physical health (Bushe & Holt, 2004; Crump, Winkleby, Sundquist, & Sundquist, 2013; Hennekens, Hennekens, Hollar, Casey, & Raton, 2005; Osborn et al., 2008), and associated health behaviours including smoking, poor diet and reduced physical activity (McCreadie, 2002, 2003).

In addition, cognitive impairment, a core component of schizophrenia (Bora, 2015), could increase risk for dementia via reduced cognitive or brain reserve (Barnett, Salmond, Jones, & Sahakian, 2006), whereby those with a lower level of baseline cognitive functioning may require less neuropathology before meeting the clinical threshold for dementia diagnosis (Stern, 2006). Although these pathways may be more plausible in relation to chronic schizophrenia, they could also apply to those with VLOSLP, some of whom may have had longstanding subthreshold psychotic symptoms or schizotypal traits prior to VLOSLP diagnosis (Kay & Roth, 1961). In addition, stress and shared personality factors, including high levels of neuroticism, could partially account for these associations, having been identified as predictors of both schizophrenia (Howes et al., 2004; Lonnqvist et al., 2009; Van Os & Jones, 2001) and dementia (Johansson et al., 2010; Sindi et al., 2017; Terracciano et al., 2021). The relationship between VLOSLP and dementia could also reflect shared genetic vulnerabilities (Lyketsos & Peters, 2015), although a recent study using data from the English Longitudinal Study of Ageing found that, among community-dwelling adults aged >50 years, polygenic score for schizophrenia was associated with cognitive impairment at baseline, but not cognitive decline over 10 years of follow-up (Kępińska et al., 2020).

Conversely, it is possible that VLOSLP symptoms could represent an early marker of Alzheimer's disease neuropathology, given that amyloid-*β* has been found to accumulate in the brain over several decades before dementia onset (Villemagne et al., 2013), and these neuropathological changes may lead to emergence of cognitive and non-cognitive symptoms of dementia prior to diagnostic threshold being reached. In line with this, the concept of mild behavioural impairment posits that late-onset neuropsychiatric symptoms, including apathy, emotion dysregulation, reduced impulse control, agitation, social inappropriateness, and psychotic symptoms, reflect possible early markers of preclinical dementia neuropathology (Ismail et al., 2017).

Further, depression has consistently been found to be associated with subsequent dementia (Diniz, Butters, Albert, Dew, & Reynolds, 2013; Ownby, Crocco, Acevedo, John, & Loewenstein, 2007), and several cohort studies have demonstrated stronger associations between late-onset depression and dementia, relative to early or mid-life depression (Heser et al., 2013; Karlsson et al., 2015; Li et al., 2011). A recent cohort study with 28 years of follow-up demonstrated that the association between dementia and depression symptoms became apparent 11 years before dementia diagnosis, potentially reflecting prodromal dementia, albeit more than a decade before diagnosis (Singh-Manoux et al., 2017). It is clear that psychiatric symptoms with a late age-at-onset, including psychosis, are a marker of increased risk of dementia during the pre-cognitive impairment phase, and this group may be a priority group for future early intervention research in dementia (Sperling et al., 2011). Furthermore, while antipsychotic medication is effective for psychosis symptoms in people with VLOSLP and well-tolerated (Howard et al., 2018), further attention may be required on the long-term effects to guide antipsychotic use, considering the potential for adverse effects of antipsychotic use in dementia (Wang et al., 2005).

## Conclusions

In this nationally representative sample, we found a strong and persistent association between very late-onset psychotic disorders and dementia. Our findings indicate that further investigation of the potential pathways between psychosis and dementia is warranted to fully understand and characterise how psychiatric morbidities accumulate together to perpetuate disparities in mental health disorders across the life course, and to inform future management approaches.

## Supplementary material

For supplementary material accompanying this paper visit https://doi.org/10.1017/S0033291721002099. click here to view supplementary material
