# Supplementary material for: Association between risk of dementia and very late-onset schizophrenia-like psychosis: a Swedish population-based cohort study
Source: Psychol Med. 2021 May 25;53(3):750–8. doi: 10.1017/S0033291721002099 (PMC9975996; doi:10.1017/S0033291721002099)
Supplement: Supplementary file 1 [file S0033291721002099sup001.docx]

**Supplementary materials**
**Supplementary Table 1-** Very late-onset schizophrenia-like psychosis diagnostic codes **Supplementary Table 2-** Dementia diagnostic codes
**Supplementary Table 3-** Proportion of full cohort (N=3,077,366) with missing data on disposable income at age 60 or educational attainment, according to other characteristics
**Supplementary Table 4-** Assessment of proportional hazards assumption **Supplementary Table 5**- Effect modification between very late-onset schizophrenia-like psychosis and sex, familial liability of non-affective psychotic disorder, and educational attainment
**Supplementary Table 6-** Comparison of distributions for baseline survivorship (model fit assessed via AIC) **Supplementary Table 7**- Cox regression mortality hazard ratios by very late-onset schizophrenia-like psychosis group

**Supplementary Table 1- Very late-onset schizophrenia-like psychosis diagnostic codes**

| ICD-10 codes  1997 onwards | Diagnostic classification system  ICD-9 codes  1987-1996 | ICD-8 codes 1969-1986 |
| --- | --- | --- |
| F20.0: Paranoid schizophrenia F20.1: Disorganised schizophrenia F20.2: Catatonic schizophrenia F20.3: Undifferentiated schizophrenia F20.5: Residual schizophrenia F20.8: Other schizophrenia F20.9: Schizophrenia, unspecified  F21: Schizotypal disorder F23: Brief psychotic disorder F24: Shared psychotic disorder F25: Schizoaffective disorder F28: Other psychotic disorder not due to a substance or known physiological condition F29: Unspecified psychosis not due to a substance or known physiological condition | 295A: Schizophrenia, simple type  295B: Schizophrenia, disorganised type 295C: Schizophrenia, catatonic type 295D: Schizophrenia, paranoid type 295E: Acute schizophrenic episode 295F: Borderline schizophrenic condition 295G: Chronic undifferentiated schizophrenia 295H: Schizoaffective type 295W: Other specified form of schizophrenia 295X: Schizophrenia, unspecified 297A: Paranoid state, simple 297B: Delusional disorder 297C: Paraphrenia 297D: Induced psychosis 297W: Other specified paranoid conditions 297X: Paranoid condition, unspecified 298C: Reactive state of confusion 298E: Reactive paranoid psychosis 298W: Other specified reactive psychosis 298X: Reactive psychosis, unspecified | 295.0: Schizophrenia, simple type 295.1: Schizophrenia, hebephrenic type 295.2: Schizophrenia, catatonic type 295.3: Schizophrenia, paranoid type 295.4: Acute schizophrenic episode 295.5: Latent schizophrenia 295.6: Residual schizophrenia 295.7: Schizoaffective type 295.8: Other specified type of schizophrenia 295.9: Unspecified schizophrenia  297.0: Paranoid state, simple 297.1: Paranoia 297.2: Paraphrenia 297.3: Induced psychosis 297.8: Other specified paranoid state 297.9: Unspecified paranoid state 298.2: Reactive confusion 298.3: Acute paranoid reaction 298.4: Psychogenic paranoid psychosis 298.8: Other unspecified reactive psychosis 298.9: Unspecified psychosis |

**Supplementary Table 2- Dementia diagnostic codes**

| ICD-10 codes 1997 onwards | Classification system ICD-9  1987-1996 | ICD-8  1969-1986 |
| --- | --- | --- |
| F00.1: Late onset Alzheimer’s disease F00.2: Atypical/mixed Alzheimer’s disease F00.9: Unspecified Alzheimer’s disease F01: Vascular dementia F02: Dementia in other diseases classified elsewhere F03: Unspecified dementia G30: Alzheimer’s disease  G31.1: Senile degeneration of brain, not elsewhere classified G31.8: Lewy body dementia | 290A: Senile dementia 290B: Presenile dementia 290E: Multi-infarct dementia 290W: Other specified senile dementia 290X: Dementia associated with aging, unspecified 291C: Alcohol-related dementia 294B: Dementia in somatic disease classified elsewhere 331A: Presenile and senile Alzheimer’s degeneration 331B: Pick’s disease | 290.0: Senile dementia, simple type 290.1: Presenile dementia 290.2: Senile dementia, depressed or paranoid type 290.3: Senile dementia with acute confusional state 290.4: Arteriosclerotic dementia 290.8: Other senile and presenile organic psychotic conditions 290.9: Unspecified senile and presenile organic psychotic conditions |

**Supplementary Table 3- Proportion of full cohort (N=3,077,366) with missing data on disposable income at age 60 or educational attainment, according to other characteristics**

| **Variable** | **Missing, N (%)** | **χ^2^ p-value** | |
| --- | --- | --- | --- |
| **Dementia** | 4,028 (2.94%) | | p<.001 |
| No dementia | 158,318 (5.52%) | |  |
| **VLOSLP** | 1,579 (9.00%) | | p<.001 |
| No VLOSLP | 160,767 (5.38%) | |  |
| **Sex** |  | | p<.001 |
| Men | 92,819 (6.22%) | |  |
| Women | 69,527 (4.59%) | |  |
| **Familial liability of non-affective psychotic disorder** | 3,375 (4.80%) | | p<.001 |
| No familial liability of non-affective psychotic disorder | 158,971 (5.41%) | |  |
| **Region of birth** |  | |  |
| Sweden | 106,933 (4.05%) | | p<.001 |
| Finland | 7,182 (6.13%) | |  |
| Other European | 21,486 (15.76%) | |  |
| Other Nordic | 10,659 (18.94%) | |  |
| Other | 16,086 (28.41%) | |  |

**Supplementary Table 4- Assessment of proportional hazards assumption**

| **Variable** | **Schoenfeld residuals test**^a^ |
| --- | --- |
| **VLOSLP (ref: no VLOSLP)** | **χ^2^(1)=774.61, p<.001** |
| **Sex** |  |
| Female (ref: male) | χ^2^(1)=0.99, p=0.32 |
| **Familial liability of non-affective psychotic disorder (ref: none)** | χ^2^(1)=3.13, p=0.08 |
| **Educational attainment (ref: high school education)** |  |
| Pre-high school education | χ^2^(1)=0.21, p=0.65 |
| Post-high school education | χ^2^(1)=0.00, p=0.99 |
| **Disposable income at age 60 (ref: Quartile 4 (highest)** |  |
| Income quartile 3 | χ^2^(1)=3.34, p=0.07 |
| Income quartile 2 | χ^2^(1)=1.37, p=0.24 |
| Income quartile 1 (lowest) | χ^2^(1)=0.01, p=0.94 |
| **Region of birth (ref: Sweden)** |  |
| Finland | χ^2^(1)=1.18, p=0.28 |
| Other European | χ^2^(1)=0.08, p=0.78 |
| Other Nordic | χ^2^(1)=6.17, p=0.01 |
| Other | χ^2^(1)=0.16, p=0.69 |

| **Variable** | **Comparison group^a^ HR, 95%CI** | **VLOSLP group^a^ HR, 95%CI** | **Likelihood ratio test p-value for interaction** |
| --- | --- | --- | --- |
| **Sex** |  |  | P<.001 |
| Men | Ref | Ref |  |
| Women | 1.10 (1.05 – 1.14) | 0.86 (0.79 – 0.92) |  |
| **Familial liability of non-affective psychotic disorder** | | | P=0.01 |
| No familial liability of psychotic disorder | Ref | Ref |  |
| Familial liability of psychotic disorder | 1.21 (1.09 – 1.35) | 0.94 (0.80 – 1.11) |  |
| **Educational attainment** | | | P<.001 |
| High school | Ref | Ref |  |
| Pre-high school | 1.03 (0.99 – 1.08) | 0.80 (0.73 – 0.87) |  |
| Post-high school | 0.93 (0.86 – 0.99) | 0.98 (0.85 – 1.12) |  |

**Supplementary Table 5- Effect modification between very late-onset schizophrenia-like psychosis and sex, familial liability of non-affective psychotic disorder, and educational attainment**

^a^VLOSLP group, sex, education level, offspring non-affective psychotic disorder, disposable income at age 60, region of birth and matching variable

Supplementary Table 6- Comparison of distributions for baseline survivorship (model fit assessed via Akaike’s Information Criterion)

| **Distribution** | **AIC^a^** |
| --- | --- |
| Exponential | 106823.9 |
| Weibull | 106817.1 |
| Lognormal | 108391.8 |
| Loglogistic | 106858.9 |
| ^a^Adjusted for: VLOSLP group, sex, education level, offspring non-affective psychotic disorder, disposable income at age 60, region of birth and matching variable. Lower AIC values indicated a better fit to the data | |

|  |
| --- |

**Supplementary Table 7****- Cox regression mortality hazard ratios by very late-onset schizophrenia-like psychosis group**

| **Variable** | **Mortality HR  (95%CI) Adj1** | **Mortality HR (95%CI) Adj2** |
| --- | --- | --- |
| **VLOSLP (ref: no VLOSLP)** | 2.83 (2.77 – 2.89) | 2.85 (2.78 – 2.91) |
| **Adj 1:** Matching variable **Adj2:** VLOSLP group, sex, education level, offspring non-affective psychotic disorder, disposable income at age 60, region of birth and matching variable | | |
